# Human perforin gene variation is geographically distributed

**DOI:** 10.1002/mgg3.344

**Published:** 2017-12-07

**Authors:** Robin C. Willenbring, Yasuhiro Ikeda, Larry R. Pease, Aaron J. Johnson

**Affiliations:** ^1^ Mayo Clinic Graduate School of Biomedical Sciences College of Medicine Mayo Clinic Rochester MN USA; ^2^ Department of Immunology Mayo Clinic Rochester MN USA; ^3^ Department of Molecular Medicine Mayo Clinic Rochester MN USA; ^4^ Department of Neurology Mayo Clinic Rochester MN USA

**Keywords:** familial hemophagocytic lymphohistiocytosis type 2, human genetics, pathogen, perforin

## Abstract

**Background:**

Deleterious mutations in *PRF1* result in lethal, childhood disease, familial hemophagocytic lymphohistiocytosis type 2 (FHL 2). However, not all mutations in *PRF1* are deleterious and result in FHL 2. Currently, these nondeleterious mutations are being investigated in the onset of numerous disorders, such as lymphomas and diabetes. Yet, there is still an overwhelmingly large amount of *PRF1* mutations that are not associated with disease.

**Methods:**

We conducted a post hoc analysis of the *PRF1* mutations in the coding region using the recently published Exome Aggregation Consortium genomes, Leiden Open Variation Database, NCBI SNP database, and primary literature to better understand *PRF1* variation in the human population.

**Results:**

This study catalogs 460 *PRF1* mutations in the coding region, and demonstrates *PRF1* is more variant then previously predicted. We identify key *PRF1* mutations with high allelic frequency and are only found in certain populations. Additionally, we define *PRF1* SNVs are geographically distributed.

**Conclusions:**

This study concludes with a novel hypothesis that nondeleterious mutation in *PRF1*, which decreases perforin expression and/or activity, may be an example of selective advantage in the context of environmental stressors prevalent near the equator. Our studies illustrate how perforin deficiency can be protective from injuries resulting in blood–brain barrier (BBB) disruption.

## 1 INTRODUCTION

Perforin is a pore‐forming protein expressed by cytotoxic CD8 T cells and natural killer (NK) cells and is required for cell‐mediated cytotoxicity and effective control of pathogens (van Dommelen et al., 2006; Tschopp & Nabholz, 1990; Voskoboinik, Smyth, & Trapani, 2006). Deleterious mutations in the perforin gene, *PRF1*, result in a lethal childhood disease called familial hemophagocytic lymphohistiocytosis type 2 (FHL 2) (Stepp et al., 1999). FHL 2 results in ineffective virus clearance and chronic inflammation that is treated with a bone marrow transplant (Risma & Jordan, 2012). However, there also are nondeleterious mutations in *PRF1* present at measurable frequency, which reduce perforin activity, that do not necessarily result in FHL 2 (Zur Stadt et al., 2004). The biological relevance of the natural heterogeneity in the human perforin gene is not understood. One leading hypothesis is that specific nondeleterious perforin mutations predispose individuals to other immunodeficient or autoimmune diseases called perforinopathies (Brennan, Chia, Trapani, & Voskoboinik, 2010; Voskoboinik & Trapani, 2013; Voskoboinik et al., 2007). Currently, perforin mutations are being investigated in the onset of lymphomas, autoimmune lymphoproliferative syndrome (ALPS), and acquired aplastic anemia (Brennan et al., 2010; Buttini et al., 2015; Cappellano et al., 2008; Clementi et al., 2006; Feldmann et al., 2005; Revelo et al., 2014; Voskoboinik & Trapani, 2013). Yet, this hypothesis does not explain why healthy individuals often have perforin mutations.

Another possibility is that perforin variants exist in the population as a consequence of selective advantage during certain kinds of inflammation. Perforin is critical for virus and intracellular bacteria clearance. Perforin is also required for cytotoxic activity against tumors and may play a role in immune surveillance (Pipkin & Lieberman, 2007). However, perforin has also been demonstrated to be a key modulator of pathologic blood–brain barrier (BBB) disruption during CNS viral infection and experimental cerebral malaria (Nitcheu et al., 2003; Suidan, McDole, Chen, Pirko, & Johnson, 2008). Severity of BBB disruption occurs in a perforin gene dosage‐dependent manner with higher perforin expression resulting in more disruption (Willenbring et al., 2016). Therefore, if perforin diversity is considered in regard to balancing the need to defend against infection with the attenuation of immune‐mediated pathology, for specific kinds of infection, perforin single‐nucleotide variants (SNVs) could be associated with a selection advantage.

Verifying the potential selective advantage associated with various perforin alleles has been difficult due to lack of analysis on perforin SNVs in healthy individuals. Recently, it has become possible to investigate genetic variation of perforin in a large number of characterized individuals, from geographically distinct populations. The Exome Aggregation Consortium has compiled 60,706 genomes from individuals of African, Latino, South Asian, East Asian, and European descent (Lek et al., 2016). Using these data, we investigated the genetic variation of *PRF1* modeling their impact on the perforin protein, and evaluate perforin diversity as compared to other related genes. From this work, we put forward a hypothesis and model of how perforin genetic diversity may provide of selective advantage in certain environments.

## 2 METHODS

### 2.1 Study subjects

The Broad Institute Exome Aggregation Consortium is a collaboration of investigators with the goal to aggregate and harmonize exome sequencing data from a variety of large‐scale studies, which is available to the scientific community (Lek et al., 2016). The consortium, which comprised multiancestry cohorts, includes African ancestry (AFR), Latino ancestry (LAT), South Asian ancestry (SA), East Asian ancestry (EA), European (non‐Finnish) ancestry (EUR), and Finnish ancestry (FIN) pooled, whole genome data. The sample population consists of 60,706 genomes. All human data were provided from the open access databases, Leiden Open Variation Database (LOVD), Exome Aggregation Consortium database (ExAC), and NCBI SNP database (An, Gursoy, Gurgey, & Keskin, 2013; Clementi et al., 2006; Feldmann et al., 2002; Goransdotter Ericson et al., 2001; Horne et al., 2008; Husami, 2011; Lee et al., 2006; Lek et al., 2016; Stepp et al., 1999; Urrea Moreno et al., 2009; Voskoboinik, Thia, & Trapani, 2005; Zhang et al., 2011; Zur Stadt et al., 2006).

### 2.2 Proteomic structure of perforin

Perforin protein structure was illustrated using PyMOL (pymol.org, Schrodinger, LLC). The 3NSJ file was uploaded into PyMOL and used to demonstrate perforin structure and diversity among subdomains. Structural data were from the NCBI Protein Database, originally published in 2010 (Law et al., 2010).

### 2.3 Compiling perforin SNVs

All perforin SNVs were compiled using the Exome Aggregation Consortium Database, the Leiden Open Variation Database, and primary literature reporting human perforin mutations. Only SNVs in the coding region of perforin were used in this study. When comparing genomic diversity to other genes, only Exome Aggregation Consortium Database data were used for perforin and all other genes investigated.

### 2.4 Identifying perforin mutation classes associated with geographical regions

To define which perforin mutations should be described in reference to the frequency in distinct ethnic groups, we first identified the missense mutations within the pooled ExAC database that had an allelic frequency >0.001. Additionally, mutations of high interest based on their prevalence in the literature and association with disease were also included (Lee et al., 2006; Voskoboinik et al., 2005). This resulted in a list of 33 mutations. From this list, we then identified mutations that are predominant in one particular ethnic group, and/or have a previously reported defined effect on perforin activity. It should be noted that many perforin mutations have not been tested to determine their effect on function and still remain undefined.

### 2.5 Calculating genomic diversity

For the purposes of this manuscript, all mutations in the coding region of human perforin gene (*PRF1*) are taken into consideration to better understand genetic diversity or genetic variation. To determine the genomic diversity of *PRF1* compared to other human genes, we calculated the single‐nucleotide variant rate (SNVR), missense mutation rate (MR), and variance using the following equations:

(1) SNVR=a/b

*a *= number of different SNVs in the coding region of the gene

*b *= number of nucleotides in coding region of the gene

(2) MR=c/d

*c *= number of missense mutations

*d *= number of amino acids in protein

(3) $$\begin{array}{cc} & \text{Variance of multiple genes within a population}\\ & =\sum \left(\frac{a}{a-1}\right)\left(\frac{{\left(\sigma_{n}\;\times a\right)}_{n}}{b}\right) \end{array}$$

*a = *number of SNVs in the coding region of the gene

*n = *a given SNV

σ_*n*_ = allelic frequency of a given SNV

*b* = number of nucleotides in coding region of the gene

(4) $$\begin{array}{cc} & \text{Variance of one gene between different populations}\\ & =\frac{\sum \left(\frac{a}{a-1}\right)\left(\frac{{\left(\sigma_{n}\;\times a\right)}_{n}}{b}\right)}{z} \end{array}$$

*a = *number of SNVs in the coding region of the gene

*n = *a given SNV

σ_*n *_= allelic frequency of a given SNV

*b* = number of nucleotides in coding region of the gene

*z *= number of alleles for a given gene in the population

The variance of a gene calculation is based off of Tajima's D equation to include both the amount of mutations in the coding region of the gene and the allelic frequency of each mutation.

### 2.6 Defining equatorial distance

Equatorial distance was determined through use of latitude degrees. Genomic data were divided into six ancestral regions. These regions are Africa, South Asia, East Asia, Latin America, Europe non‐Finland, and Finland. Ancestral ethnicity was associated with each region as defined by the ExAC browser database. For each of these regions, a representative latitude degree was assigned for basis of discerning equatorial distance. The representative latitude was determined by choosing the median latitude for the defined region.

### 2.7 Statistical analysis

All graphs were made using GraphPad Prism (Graphpad Software, San Diego, CA). Statistical tests were run in GraphPad Prism Statistical Software (GraphPad Software).

## 3 RESULTS

### 3.1 Ratio of missense mutations to amino acids is highest in amphipathic α‐helix

**Figure 1:**
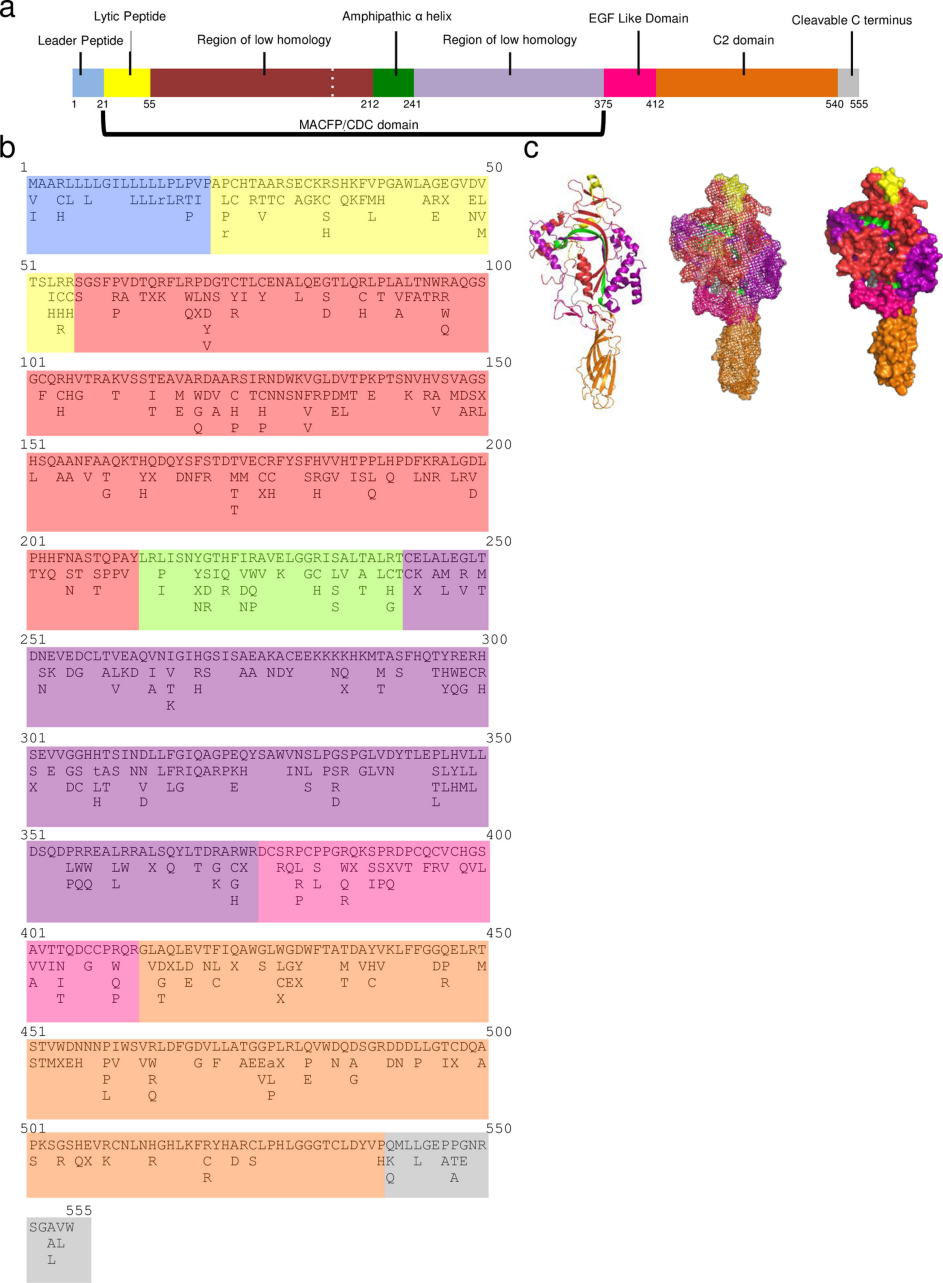
Perforin mutations are present in all domains and subdomains of this pore‐forming protein. (a) Genomic organization of *PRF1* with indicated domains and subdomain. (b) List of amino acids, using single letter code, with corresponding mutations. Lower case letters indicate that there is a frameshift or deletion that results in an amino acid termination later in the protein. An “X” indicated a termination at that amino acid (c) 3D rendering (cartoon, mesh, surface) of perforin structure

Human perforin is 555 amino acids, has a molecular weight of 67 kDa, and is organized into three major domains: the membrane attack complex perforin‐like/cholesterol‐dependent cytolysin (MACPF/CDC) domain, the epidermal growth factor (EGF) domain, and the C terminus domain. These three domains are further organized into eight subdomains (Figure 1a,c). We have determined using the ExAC Browser database and primary literature sources that there are 460 SNVs present in the coding region of human *PRF1* (Figure 1b) (An et al., 2013; Clementi et al., 2006; Feldmann et al., 2002; Goransdotter Ericson et al., 2001; Horne et al., 2008; Husami, 2011; Lee et al., 2006; Lek et al., 2016; Stepp et al., 1999; Urrea Moreno et al., 2009; Voskoboinik et al., 2005; Zhang et al., 2011; Zur Stadt et al., 2006). Of the 555 amino acids in perforin, over half are associated with a mutation. Missense mutations account for 350 of the 460 SNVs (Table 1).

**Table 1:** Summary of perforin SNVs broken down by subdomain. Each subdomain lists the total number of SNVs, mutations, amino acids in that subdomain, and the ratio of missense mutations to amino acids

Perforin domains	Number of SNVs	Number of missense mutations	Total amino acids	Ratio of missense mutations to amino acids
Leader peptide	15	9	21	0.429
Lytic peptide	37	29	34	0.853
Region of low homology I	132	100	157	0.571
Amphipathic α‐helix	32	28	29	0.966
Region of low homology II	121	78	134	0.582
EGF‐like domain	37	28	37	0.757
C2 domain	76	61	128	0.477
Cleavable C‐terminus	10	7	15	0.467
Total	460	340	555	0.613 (average)

We next established which subdomain of perforin contained the highest ratio of missense mutations to amino acids (Table 1). The amphipathic α‐helix has the highest ratio of missense mutations to amino acids compared to other perforin subdomains with a ratio of 0.966 (Figure 1, Table 1). Not all amino acids within the amphipathic α‐helix are associated with missense mutations. However, there are residues within this subdomain that are associated with multiple missense mutations (Figure 1, Table 1). The lytic peptide subdomain has the next highest ratio of missense mutations to amino acids, with a ratio of 0.853 (Table 1). In contrast, the leader peptide, C2 domain, and cleavable C terminus have the lowest missense mutations to amino acid ratios of 0.477 and 0.467, respectively (Figure 1, Table 1). These data demonstrate the difference in the ratio of missense mutations to amino acids among different perforin subdomains, with the amphipathic α‐helix subdomain having the highest ratio.

### 3.2 Specific perforin mutations are associated with certain populations

**Figure 2:**
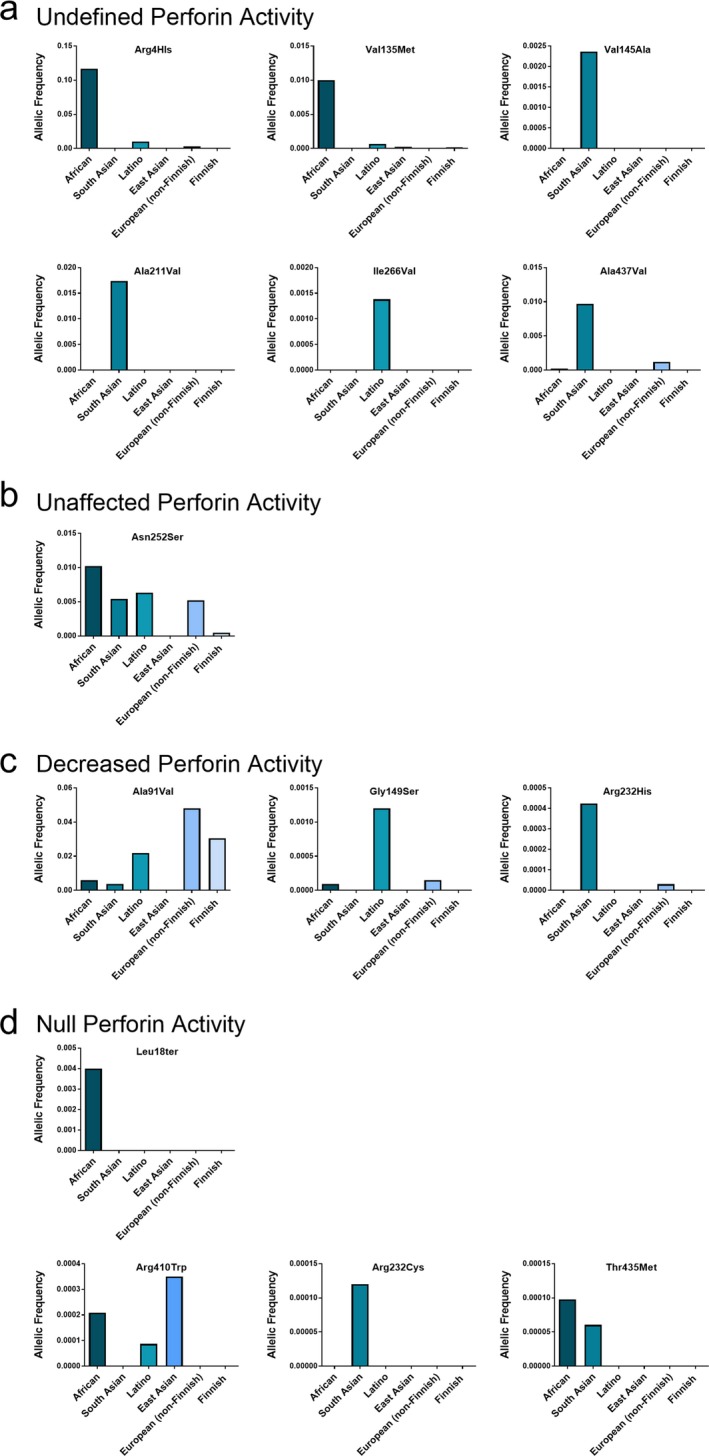
Classes of perforin mutations in association with different populations. Allelic frequencies of missense mutations, with indicated amino acid substitution, in African, South Asian, Latino, East Asian, European (non‐Finnish), and Finnish populations that have (a) undefined, (b) unaffected, (c) decreased, or (d) null perforin activity

Due to large amount of perforin mutations, we wanted to determine if certain classes of perforin mutations were associated with geographical regions. Using the ExAC browser database we were able to investigate the allelic frequency of certain perforin mutations associated with different populations (see Section Methods). From our identification criteria, we identified 33 perforin mutations of interest (data not shown). From this list of 33, we investigated the allelic frequency of 14 perforin mutations (Figure 2). Of these 14 perforin mutations, six did not have a defined perforin activity associated with them. However, these mutations nonetheless had a higher frequency in certain populations (Figure 2a). The missense mutations Arg4His and Val135Met are most frequent in the African population (Figure 2a), although the missense mutations causing Val145Ala, Ala211Val, and Ala437Val substitutions are most frequent in the South Asian population (Figure 2a), the amino acid substitution Ile266Val is most frequent in the Latino population (Figure 2a).

A missense mutation that does not affect perforin activity, Asn252Ser, is present at comparable allelic frequency in four of the six populations studied (Figure 2b). The perforin amino acid substitution Ala91Val is well documented and results in reduced perforin activity (Voskoboinik et al., 2005, 2007). This mutation had the highest allelic frequency in European (non‐Finnish) and Finnish populations (Figure 2c). Other missense mutations that reduce perforin activity are Gly149Ser and Arg232His. These substitutions have the highest allelic frequency in the Latino and South Asian populations, respectively (Figure 2c) (Risma, Frayer, Filipovich, & Sumegi, 2006). Perforin mutations that result in null perforin activity have the highest allelic frequency in the African, South Asian, and East Asian population (Figure 2d). In the list of perforin mutations investigated, deleterious mutations were not detected in European (non‐Finnish) and Finnish populations (data not shown, Figure 2d).

### 3.3 Perforin allelic diversity is comparable to HLA molecules

**Figure 3:**
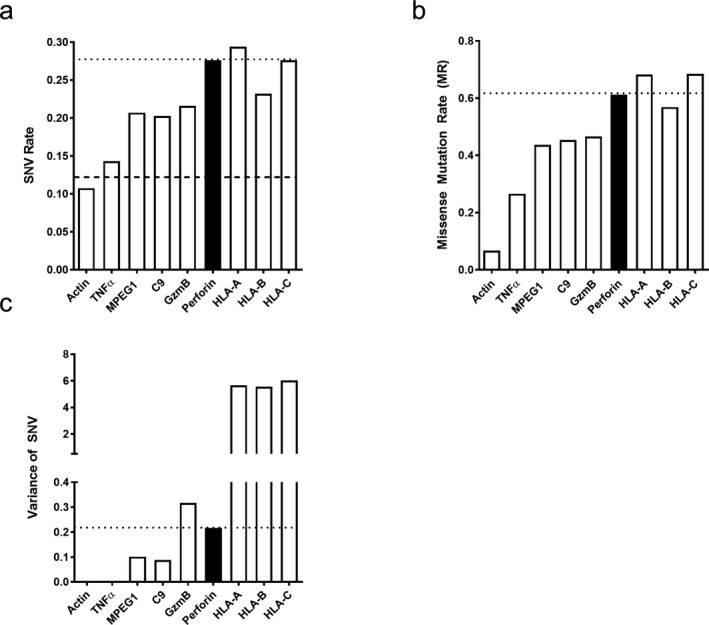
Perforin variability is higher than other genes but not as high as class I genes. Perforin (a) SNV rate, (b) mutation rate, and (c) variance as measured by Tajima's D compared to other genes

The variant nature of perforin prompted us to compare perforin allelic diversity to other, relevant molecules. First, we determined the SNV rate for the genes encoding β‐actin (actin), tumor necrosis factor alpha (TNFα), perforin‐2 (MPEG1), complement 9 (C9), granzyme B (GzmB), human leukocyte antigens A, B, and C (HLA‐A,B,C), and perforin (Figure 3a). SNV rate is defined as the number of mutations in the coding region of the protein divided by the total number of nucleotides in the coding region of the protein (see Section Methods). As expected, the HLA genes had the highest SNV rate. Also as expected, the highly conserved gene encoding actin had the lowest SNV rate among genes we analyzed. Similarly, the proinflammatory cytokine, TNFα, also had a low SNV rate. In contrast, perforin had a high SNV rate, comparable to HLA molecules (Figure 3a). Meanwhile, the gene encoding C9, the most homologous protein to perforin, had an intermediate SNV rate, as does the GzmB and MPEG1 gene.

The presence of a SNV in a specific gene does not necessarily result in an amino acid change. Therefore, we next determined the missense mutation rate of each of the genes in our panel. Missense mutation rate (MR) was calculated by taking the number of missense mutations present in the coding region divided by the number of amino acids present in the protein (see Section Methods). Consistent with our calculated SNV rate, actin and TNFα MRs were the lowest. The MR of C9, MPEG1, and GzmB was intermediate. Meanwhile, the MR of perforin was high and comparable to HLA‐A, B, and C (Figure 3b).

The SNV rate and MR rate inform us on the presence of diversity in the perforin gene, but do not reflect the prevalence of variants within the population. To define prevalence of variants in the human population, we determined the SNV variance for each aforementioned gene using Tajima's D equation (Tajima, 1989). We apply Tajima's D equation to combine the amount of variants present in the gene and the allelic frequency of each variant to evaluate variance (Tajima, 1989). As expected, the genes for the HLA molecules had a very high level of variance. Furthermore, the genes encoding actin and TNFα had very low SNV variance. The other genes studied that encode for C9, GzmB, MPEG1, and perforin all had an intermediate level of variance as determined by Tajima's D equation (Figure 3c). Although not as high as the HLA molecules, perforin SNV variance was considerably higher than actin, TNFα, MPEG1, and C9. A molecule found in the cytotoxic granule with perforin, GzmB, has almost threefold higher variance than C9 and slightly higher variance than PRF1 (Figure 3c). Perforin genetic variance did not surpass GzmB or HLA gene diversity, but is still higher than other molecules investigated (Figure 3).

### 3.4 Perforin variance is indirectly correlated with distance from equator

**Figure 4:**
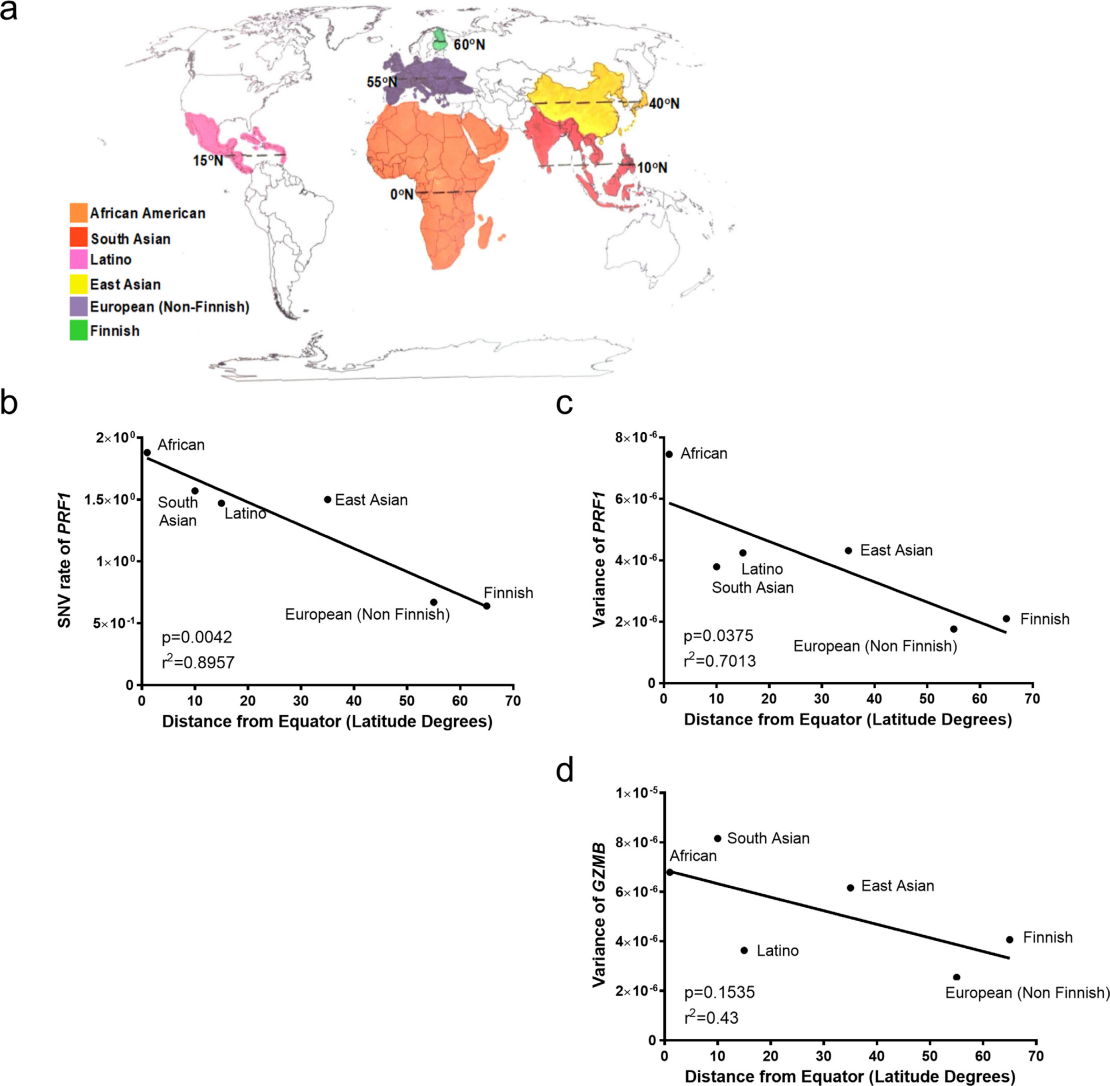
Perforin SNVs are geographically distributed. (a) Representative world map indicting equatorial distance for each population. The (a) SNV rate of perforin (*PRF1*), (b) variance of *PRF1*, and (c) variance of *GZMB* are plotted against equatorial distance. Linear regression analysis was used to test the relationship between these two values. *R* ^2^ and *p* values are indicated on graph. *p*s < .05 are considered significant

Allelic shaping pressures associated with geographically distinct regions have influenced diversity in immunological genes (Jeffery & Bangham, 2000). To determine if geographically distinct pressures could potentially shape perforin gene diversity, we investigated perforin SNV variance in populations closer and farther away from the equator. Since GzmB and perforin had comparable SNV variance, we also determined the equatorial distribution of GzmB SNV variance. Again, we applied Tajima's D to define the SNV variance for the genes encoding perforin and granzyme B for populations of African, Latino, South Asian, East Asian, European non‐Finnish, and Finnish descent. Each population had a distinct geographical region which could be assigned a distance from the equator, in latitude degrees (Figure 4a). Then using linear regression analysis, we assessed the relationship between the equatorial distance and SNV variance of perforin and GzmB for each population. As shown in Figure 4, we detected a significant, indirect correlation between *PRF1* SNV variance and equatorial distance (Figure 4c). Furthermore, we investigated the correlation between perforin SNV rate and equatorial distance using a linear regression analysis. There was a significant correlation detected between perforin SNV rate and equatorial distance (Figure 4d). While a similar relationship between granzyme B SNV variance and equatorial distance was evident, the correlation did not reach significance (Figure 4b).

## 4 DISCUSSION

In this study, we indexed 460 perforin SNVs in the coding region of the perforin gene, which is fourfold greater than previously reported (An et al., 2013). Furthermore, we categorize each subdomain of perforin as the ratio of missense mutations to amino acids. Subdomains that have a higher ratio may include subdomains that are less rigid in the amino acids necessary to be functional. The amphipathic α‐helix had the highest ratio of missense mutations to amino acids (Table 1). This domain is part of the lipid membrane‐inserting domain (Persechini et al., 1992). If a mutation still allows for the protein to be inserted into a lipid membrane, this subdomain will retain function. In contrast, subdomains with a lower ratio of missense mutations to amino acids tend to be more critical to perforin protein function. This includes the C2 domain. The C2 domain is responsible for binding calcium, which is required to maintain proper perforin function. Additionally, the leader peptide protein also had a low ratio of missense mutations to amino acids. This could be due to the leader peptide serving an important role in directing perforin toward the secretory pathway (Voskoboinik & Trapani, 2006). Additionally, it has been reported that the first 19 amino acids are necessary for proper pore formation and lytic activity (Persechini et al., 1992). Altering this subdomain could therefore be deleterious to pore formation, rendering perforin nonfunctional. Structure function studies need to be conducted to better understand the extent the ratio of missense mutations to amino acids reflects the mutation tolerance of perforin subdomains.

Since there are 460 *PRF1* SNVs, we wanted to identify mutations of interest to further investigate in future studies. These mutations included those that were frequent across all populations or may be associated with a certain population. We identified 33 perforin mutations that had an allelic frequency of >0.001 or had a defined perforin activity associated with them. Many of these mutations were not associated with a defined perforin activity. However, some perforin mutations were more prevalent in certain populations (Figure 2). Mutations that did affect perforin activity, Ala91Val, Gly149Ser, and Arg232His, had very different population distributions. Ala91Val, a mutation that has been reported to cause 50% reduction in perforin activity, has the highest allelic frequency in European (non‐Finnish) and Finnish populations (Voskoboinik et al., 2005) (Figure 2c). However, the Latino population has the highest allelic frequency for the Gly149Ser substitution. The South Asian population has the highest allelic frequency for the Arg232H substitution. Both of these missense mutations result in decreased perforin activity. Future studies should investigate if these mutations are associated with any disease or selective advantage.

Deleterious mutations in *PRF1* can lead to the lethal childhood disease FHL 2. The ExAC browser database excludes individuals with childhood diseases. Therefore, we did not expect to find many deleterious perforin mutations in the ExAC browser population. However, we did identify deleterious mutations in the population. All of these mutations were heterozygous and were not present in the European (non‐Finnish) and Finnish populations (Figure 2d). Consistent with previous studies, Leu18Ter substitution was unique to the African population (Lee et al., 2006). The substitution Arg410Trp was present in the African, Latino, and highest in the East Asian population (Figure 2d). Previously, this mutation has been associated with FHL 2 patients (Feldmann et al., 2002). However, studies should continue to investigate any consequence being heterozygous for these mutations has on long‐term health.

Defining perforin as having more mutations than previously reported led us to compare perforin variance to both related and unrelated genes. The SNV and mutation rate of perforin were comparable to HLA‐A, B, and C, which are considered some of the most polymorphic molecules known. Furthermore, it has recently been reported the SNV rate average for the genome is higher than previously expected with 1 in 8 nucleotides are associated with a change in the average human population (SNV rate = 0.125) (Lek et al., 2016). Within the perforin gene (*PRF1*), over 1 in 4 nucleotides are associated with a change in the human population (SNV rate = 0.27) (Figure 3a). When we investigated the prevalence of SNVs using Tajima's D equation, perforin SNV variance was much lower than the variance calculated for each of the genes encoding the HLA molecules (Figure 3c). However, perforin SNV variance was still higher than the other genes in our panel and is comparable to the GzmB gene. From these data, we conclude that perforin variants may have significance since they are more prevalent than what we expected based on previous predictions (Petrovski, Wang, Heinzen, Allen, & Goldstein, 2013).

The observation that perforin gene diversity is more extensive than previously thought prompts an analysis of the significance of this finding. Genetic diversity in the human immune response is important to effectively protect against extrinsic and intrinsic health threats. The most notable example is the diversity in the major histocompatibility complex (MHC) class I and class II loci (Jeffery & Bangham, 2000). The diversity of MHC class I has been tracked in parallel with the human migration pattern, starting in Africa, through Asia, across the Bering Strait, down through North America and ending in South America (Fernandez Vina et al., 2012). Furthermore, specific haplotypes of MHC class I and II have been attributed to resistance of certain pathogens (Jeffery & Bangham, 2000). Although many immune‐related molecules have been studied in reference to their diversity, perforin, the effector molecule of CD8 T and NK cells, had not been investigated.

One indication of selective pressure for SNVs is geographical distribution. It has previously been shown that pathogen richness, a major selective pressure, is correlated with equatorial distance (Cashdan, 2014). Regions that are closer to the equator have a more diverse pathogen population than regions that are farther away (Cashdan, 2014). Since perforin is crucial for controlling intracellular pathogens, we hypothesized that perforin gene diversity may be due to a selective pressure such as pathogen richness. Using a linear regression analysis, we tested the relationship between equatorial distance and perforin SNV variance (as calculated with Tajima's D). We did detect a correlation between *PRF1* SNV variance and equatorial distance (Figure 4). In contrast, we did not observe a correlation between *GZMB* variance and equatorial distance (Figure 4). These data demonstrate that the higher perforin variance we observe in populations closer to the equator may be a result of an equatorial‐associated pathogenic pressure.

**Figure 5:**
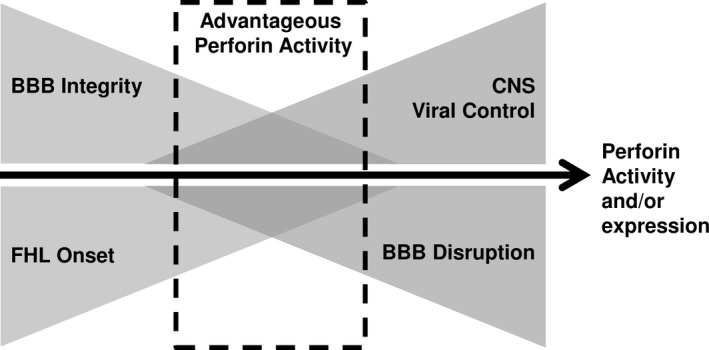
Current working model of perforin as an example of selective advantage. Based on data presented in these studies, we put forward the novel hypothesis that perforin may be a selective advantage, in regard to mediating BBB disruption and proper viral control. As perforin expression and/or activity are increased, the ability to control a viral infection is also increased. However, an individual also increases the susceptibility of having pathologic BBB disruption

Lethal pathogenic pressures could take numerous forms. In areas such as Africa, diseases that can result in CNS immune‐mediated pathology are prevalent, such as Dengue Virus Hemorrhagic Fever (DVHF), Crimean‐Congo Hemorrhagic Fever (CCHF), and cerebral malaria (CM) (Kebede, Duales, Yokouide, & Alemu, 2010). Perforin has been shown to be a key regulator of neuropathologies, such as BBB disruption in experimental models (Suidan et al., 2008). Perforin null mice are resistant to both viral‐associated BBB disruption and hemorrhage associated with cerebral malaria (Nitcheu et al., 2003; Suidan et al., 2008). However, perforin null humans suffer from lethal childhood disease, FHL2 (Stepp et al., 1999). Yet, reduction in perforin, but not ablation, reduces BBB disruption (Willenbring et al., 2016). We put forward a hypothesis that variance in perforin may contribute to maintaining the balance between immune‐mediated and pathogen‐mediated pathology (Figure 5). Perforin may be increasing the relative fitness of an individual. An individual that has a perforin mutation resulting in decreased activity and/or expression may have advantage over others in that group during a neuropathologic event due to the ability to better balance inflammation‐induced pathology. We contend that future studies should focus on the relationship between perforin mutations and protection from a perforin‐mediated pathology.

## DISCLOSURE

The authors have nothing to disclose.

## AUTHOR CONTRIBUTION

RCW, YI, LRP, and AJJ conceived and designed experiments. RCW performed the experiments. RCW, YI, LRP, and AJJ analyzed data. RCW and AJJ wrote the manuscript.

## References

[mgg3344-bib-0001] An, O. , Gursoy, A. , Gurgey, A. , & Keskin, O. (2013). Structural and functional analysis of perforin mutations in association with clinical data of familial hemophagocytic lymphohistiocytosis type 2 (FHL2) patients. Protein Science, 22(6), 823–839. https://doi.org/10.1002/pro.v22.6 23592409 10.1002/pro.2265 PMC3690721

[mgg3344-bib-0002] Brennan, A. J. , Chia, J. , Trapani, J. A. , & Voskoboinik, I. (2010). Perforin deficiency and susceptibility to cancer. Cell Death and Differentiation, 17(4), 607–615. https://doi.org/10.1038/cdd.2009.212 20075937 10.1038/cdd.2009.212

[mgg3344-bib-0003] Buttini, S. , Cappellano, G. , Ripellino, P. , Briani, C. , Cocito, D. , Osio, M. , … Comi, C. (2015). Variations of the perforin gene in patients with chronic inflammatory demyelinating polyradiculoneuropathy. Genes and Immunity, 16(1), 99–102. https://doi.org/10.1038/gene.2014.59 25354579 10.1038/gene.2014.59

[mgg3344-bib-0004] Cappellano, G. , Orilieri, E. , Comi, C. , Chiocchetti, A. , Bocca, S. , Boggio, E. , … D'alfonso, S. (2008). Variations of the perforin gene in patients with multiple sclerosis. Genes and Immunity, 9(5), 438–444. https://doi.org/10.1038/gene.2008.35 18496551 10.1038/gene.2008.35

[mgg3344-bib-0005] Cashdan, E. (2014). Biogeography of human infectious diseases: A global historical analysis. PLoS ONE, 9(10), e106752. 25271730 10.1371/journal.pone.0106752 PMC4182673

[mgg3344-bib-0006] Clementi, R. , Chiocchetti, A. , Cappellano, G. , Cerutti, E. , Ferretti, M. , Orilieri, E. , … Danesino, C. (2006). Variations of the perforin gene in patients with autoimmunity/lymphoproliferation and defective Fas function. Blood, 108(9), 3079–3084. https://doi.org/10.1182/blood-2006-02-001412 16720836 10.1182/blood-2006-02-001412

[mgg3344-bib-0007] van Dommelen, S. L. , Sumaria, N. , Schreiber, R. D. , Scalzo, A. A. , Smyth, M. J. , & Degli‐Esposti, M. A. (2006). Perforin and granzymes have distinct roles in defensive immunity and immunopathology. Immunity, 25(5), 835–848. https://doi.org/10.1016/j.immuni.2006.09.010 17088087 10.1016/j.immuni.2006.09.010

[mgg3344-bib-0008] Feldmann, J. , Le Deist, F. , Ouachee‐Chardin, M. , Certain, S. , Alexander, S. , Quartier, P. , … Fisher, A. (2002). Functional consequences of perforin gene mutations in 22 patients with familial haemophagocytic lymphohistiocytosis. British journal of haematology, 117(4), 965–972. https://doi.org/10.1046/j.1365-2141.2002.03534.x 12060139 10.1046/j.1365-2141.2002.03534.x

[mgg3344-bib-0009] Feldmann, J. , Menasche, G. , Callebaut, I. , Minard‐Colin, V. , Bader‐Meunier, B. , Le Clainche, L. , … de Saint Basile, G. (2005). Severe and progressive encephalitis as a presenting manifestation of a novel missense perforin mutation and impaired cytolytic activity. Blood, 105(7), 2658–2663. https://doi.org/10.1182/blood-2004-09-3590 15598808 10.1182/blood-2004-09-3590

[mgg3344-bib-0010] Fernandez Vina, M. A. , Hollenbach, J. A. , Lyke, K. E. , Sztein, M. B. , Maiers, M. , Klitz, W. , … Cao, K. (2012). Tracking human migrations by the analysis of the distribution of HLA alleles, lineages and haplotypes in closed and open populations. Philosophical Transactions of the Royal Society of London. Series B, Biological sciences, 367(1590), 820–829. https://doi.org/10.1098/rstb.2011.0320 22312049 10.1098/rstb.2011.0320 PMC3267126

[mgg3344-bib-0011] Goransdotter Ericson, K. , Fadeel, B. , Nilsson‐Ardnor, S. , Soderhall, C. , Samuelsson, A. , Janka, G. , … Egeler, R. M. (2001). Spectrum of perforin gene mutations in familial hemophagocytic lymphohistiocytosis. American Journal of Human Genetics, 68(3), 590–597. https://doi.org/10.1086/318796 11179007 10.1086/318796 PMC1274472

[mgg3344-bib-0012] Horne, A. , Ramme, K. G. , Rudd, E. , Zheng, C. , Wali, Y. , al‐Lamki, Z. , Gurgey, A. , Yalman, N. , Nordenskjold, M. , & Henter, J. I. (2008). Characterization of PRF1, STX11 and UNC13D genotype‐phenotype correlations in familial hemophagocytic lymphohistiocytosis. British Journal of Haematology, 143(1), 75–83. https://doi.org/10.1111/bjh.2008.143.issue-1 18710388 10.1111/j.1365-2141.2008.07315.x

[mgg3344-bib-0013] Husami, A. (2011). Perforin 1 (pore forming protein) (PRF1). CCHMC Molecular Genetics Laboratory Mutation Database. p v2.0 Build 34.

[mgg3344-bib-0014] Jeffery, K. J. , & Bangham, C. R. (2000). Do infectious diseases drive MHC diversity? Microbes and Infection, 2(11), 1335–1341. https://doi.org/10.1016/S1286-4579(00)01287-9 11018450 10.1016/s1286-4579(00)01287-9

[mgg3344-bib-0015] Kebede, S. , Duales, S. , Yokouide, A. , & Alemu, W. (2010). Trends of major disease outbreaks in the African region, 2003–2007. East African Journal of Public Health, 7(1), 20–29. 21413568 10.4314/eajph.v7i1.64672

[mgg3344-bib-0016] Law, R. H. , Lukoyanova, N. , Voskoboinik, I. , Caradoc‐Davies, T. T. , Baran, K. , Dunstone, M. A. , … Browne, K. A. (2010). The structural basis for membrane binding and pore formation by lymphocyte perforin. Nature, 468(7322), 447–451. https://doi.org/10.1038/nature09518 21037563 10.1038/nature09518

[mgg3344-bib-0017] Lee, S. M. , Sumegi, J. , Villanueva, J. , Tabata, Y. , Zhang, K. , Chakraborty, R. , … Filipovich, A. H. (2006). Patients of African ancestry with hemophagocytic lymphohistiocytosis share a common haplotype of PRF1 with a 50delT mutation. The Journal of Pediatrics, 149(1), 134–137.https://doi.org/10.1016/j.jpeds.2006.03.003 16860143 10.1016/j.jpeds.2006.03.003

[mgg3344-bib-0018] Lek, M. , Karczewski, K. J. , Minikel, E. V. , Samocha, K. E. , Banks, E. , Fennell, T. , … Tukiainen, T. (2016). Analysis of protein‐coding genetic variation in 60,706 humans. Nature, 536(7616), 285–291. https://doi.org/10.1038/nature19057 27535533 10.1038/nature19057 PMC5018207

[mgg3344-bib-0019] Nitcheu, J. , Bonduelle, O. , Combadiere, C. , Tefit, M. , Seilhean, D. , Mazier, D. , & Combadiere, B. (2003). Perforin‐dependent brain‐infiltrating cytotoxic CD8 + T lymphocytes mediate experimental cerebral malaria pathogenesis. Journal of Immunology, 170(4), 2221–2228. https://doi.org/10.4049/jimmunol.170.4.2221 10.4049/jimmunol.170.4.2221 12574396

[mgg3344-bib-0020] Persechini, P. M. , Ojcius, D. M. , Adeodato, S. C. , Notaroberto, P. C. , Daniel, C. B. , & Young, J. D. (1992). Channel‐forming activity of the perforin N‐terminus and a putative alpha‐helical region homologous with complement C9. Biochemistry, 31(21), 5017–5021. https://doi.org/10.1021/bi00136a015 1599928 10.1021/bi00136a015

[mgg3344-bib-0021] Petrovski, S. , Wang, Q. , Heinzen, E. L. , Allen, A. S. , & Goldstein, D. B. (2013). Genic intolerance to functional variation and the interpretation of personal genomes. PLoS Genetics, 9(8), e1003709 https://doi.org/10.1371/journal.pgen.1003709 23990802 10.1371/journal.pgen.1003709 PMC3749936

[mgg3344-bib-0022] Pipkin, M. E. , & Lieberman, J. (2007). Delivering the kiss of death: Progress on understanding how perforin works. Current Opinion in Immunology, 19(3), 301–308. https://doi.org/10.1016/j.coi.2007.04.011 17433871 10.1016/j.coi.2007.04.011 PMC11484871

[mgg3344-bib-0023] Revelo, X. S. , Tsai, S. , Lei, H. , Luck, H. , Ghazarian, M. , Tsui, H. , … Mark, T. (2014). Perforin is a novel immune regulator of obesity related insulin resistance. Diabetes, 64, 90–103. 25048196 10.2337/db13-1524

[mgg3344-bib-0024] Risma, K. A. , Frayer, R. W. , Filipovich, A. H. , & Sumegi, J. (2006). Aberrant maturation of mutant perforin underlies the clinical diversity of hemophagocytic lymphohistiocytosis. Journal of Clinical Investigation, 116(1), 182–192. 16374518 10.1172/JCI26217 PMC1319223

[mgg3344-bib-0025] Risma, K. , & Jordan, M. B. (2012). Hemophagocytic lymphohistiocytosis: Updates and evolving concepts. Current Opinion in Pediatrics, 24(1), 9–15. https://doi.org/10.1097/MOP.0b013e32834ec9c1 22189397 10.1097/MOP.0b013e32834ec9c1

[mgg3344-bib-0026] Stepp, S. E. , Dufourcq‐Lagelouse, R. , Le Deist, F. , Bhawan, S. , Certain, S. , Mathew, P. A. , … Kumar, V. (1999). Perforin gene defects in familial hemophagocytic lymphohistiocytosis. Science, 286(5446), 1957–1959. https://doi.org/10.1126/science.286.5446.1957 10583959 10.1126/science.286.5446.1957

[mgg3344-bib-0027] Suidan, G. L. , McDole, J. R. , Chen, Y. , Pirko, I. , & Johnson, A. J. (2008). Induction of blood brain barrier tight junction protein alterations by CD8 T cells. PLoS ONE, 3(8), e3037 https://doi.org/10.1371/journal.pone.0003037 18725947 10.1371/journal.pone.0003037 PMC2516328

[mgg3344-bib-0028] Tajima, F. (1989). Statistical method for testing the neutral mutation hypothesis by DNA polymorphism. Genetics, 123(3), 585–595. 2513255 10.1093/genetics/123.3.585 PMC1203831

[mgg3344-bib-0029] Tschopp, J. , & Nabholz, M. (1990). Perforin‐mediated target cell lysis by cytolytic T lymphocytes. Annual Review of Immunology, 8, 279–302. https://doi.org/10.1146/annurev.iy.08.040190.001431 10.1146/annurev.iy.08.040190.001431 2188665

[mgg3344-bib-0030] Urrea Moreno, R. , Gil, J. , Rodriguez‐Sainz, C. , Cela, E. , LaFay, V. , Oloizia, B. , … Risma, K. A. (2009). Functional assessment of perforin C2 domain mutations illustrates the critical role for calcium‐dependent lipid binding in perforin cytotoxic function. Blood, 113(2), 338–346. 18927437 10.1182/blood-2008-08-172924 PMC2615650

[mgg3344-bib-0031] Voskoboinik, I. , Smyth, M. J. , & Trapani, J. A. (2006). Perforin‐mediated target‐cell death and immune homeostasis. Nature Reviews Immunology, 6(12), 940–952. https://doi.org/10.1038/nri1983 10.1038/nri1983 17124515

[mgg3344-bib-0032] Voskoboinik, I. , Sutton, V. R. , Ciccone, A. , House, C. M. , Chia, J. , Darcy, P. K. , … Trapani, J. A. (2007). Perforin activity and immune homeostasis: The common A91V polymorphism in perforin results in both presynaptic and postsynaptic defects in function. Blood, 110(4), 1184–1190. https://doi.org/10.1182/blood-2007-02-072850 17475905 10.1182/blood-2007-02-072850

[mgg3344-bib-0033] Voskoboinik, I. , Thia, M. C. , & Trapani, J. A. (2005). A functional analysis of the putative polymorphisms A91V and N252S and 22 missense perforin mutations associated with familial hemophagocytic lymphohistiocytosis. Blood, 105(12), 4700–4706. https://doi.org/10.1182/blood-2004-12-4935 15755897 10.1182/blood-2004-12-4935

[mgg3344-bib-0034] Voskoboinik, I. , & Trapani, J. A. (2006). Addressing the mysteries of perforin function. Immunology and Cell Biology, 84(1), 66–71. https://doi.org/10.1111/icb.2006.84.issue-1 16405653 10.1111/j.1440-1711.2005.01409.x

[mgg3344-bib-0035] Voskoboinik, I. , & Trapani, J. A. (2013). Perforinopathy: A spectrum of human immune disease caused by defective perforin delivery or function. Frontiers in Immunology, 4, 441. 24376445 10.3389/fimmu.2013.00441 PMC3860100

[mgg3344-bib-0036] Willenbring, R. C. , Jin, F. , Hinton, D. J. , Hansen, M. , Choi, D. S. , Pavelko, K. D. , & Johnson, A. J. (2016). Modulatory effects of perforin gene dosage on pathogen‐associated blood‐brain barrier (BBB) disruption. Journal of Neuroinflammation, 13(1), 222 https://doi.org/10.1186/s12974-016-0673-9 27576583 10.1186/s12974-016-0673-9 PMC5006384

[mgg3344-bib-0037] Zhang, J. , Fu, R. , Wang, J. , Li, L. J. , Song, J. , Qu, W. , … Quan, J. (2011). Perforin gene mutations in patients with acquired severe aplastic anemia. Zhongguo Shi Yan Xue Ye Xue Za Zhi, 19(2), 431–434. 21518502

[mgg3344-bib-0038] Zur Stadt, U. , Beutel, K. , Kolberg, S. , Schneppenheim, R. , Kabisch, H. , Janka, G. , & Hennies, H. C. (2006). Mutation spectrum in children with primary hemophagocytic lymphohistiocytosis: Molecular and functional analyses of PRF1, UNC13D, STX11, and RAB27A. Human Mutation, 27(1), 62–68. https://doi.org/10.1002/(ISSN)1098-1004 16278825 10.1002/humu.20274

[mgg3344-bib-0039] Zur Stadt, U. , Beutel, K. , Weber, B. , Kabisch, H. , Schneppenheim, R. , & Janka, G. (2004). A91V is a polymorphism in the perforin gene not causative of an FHLH phenotype. Blood, 104(6), 1909; author reply 1910. https://doi.org/10.1182/blood-2004-02-0733 15342365 10.1182/blood-2004-02-0733

